# Supplementary material for: Chromosome-Aware Phylogenomics of Assassin Bugs (Hemiptera: Reduvioidea) Elucidates Ancient Gene Conflict
Source: Mol Biol Evol. 2023 Jul 26;40(8):msad168. doi: 10.1093/molbev/msad168 (PMC10411492; doi:10.1093/molbev/msad168)

Supplementary Fig S1. Individual topologies of each analysis from Fig 2. Branches are colored based on the current subfamiliar classification of the 106 ingroup taxa. Values at nodes show SH-aLRT, UFBoot, and sCF support values (IQ-TREE-based analyses) or local posterior probability (Astral-based analyses).

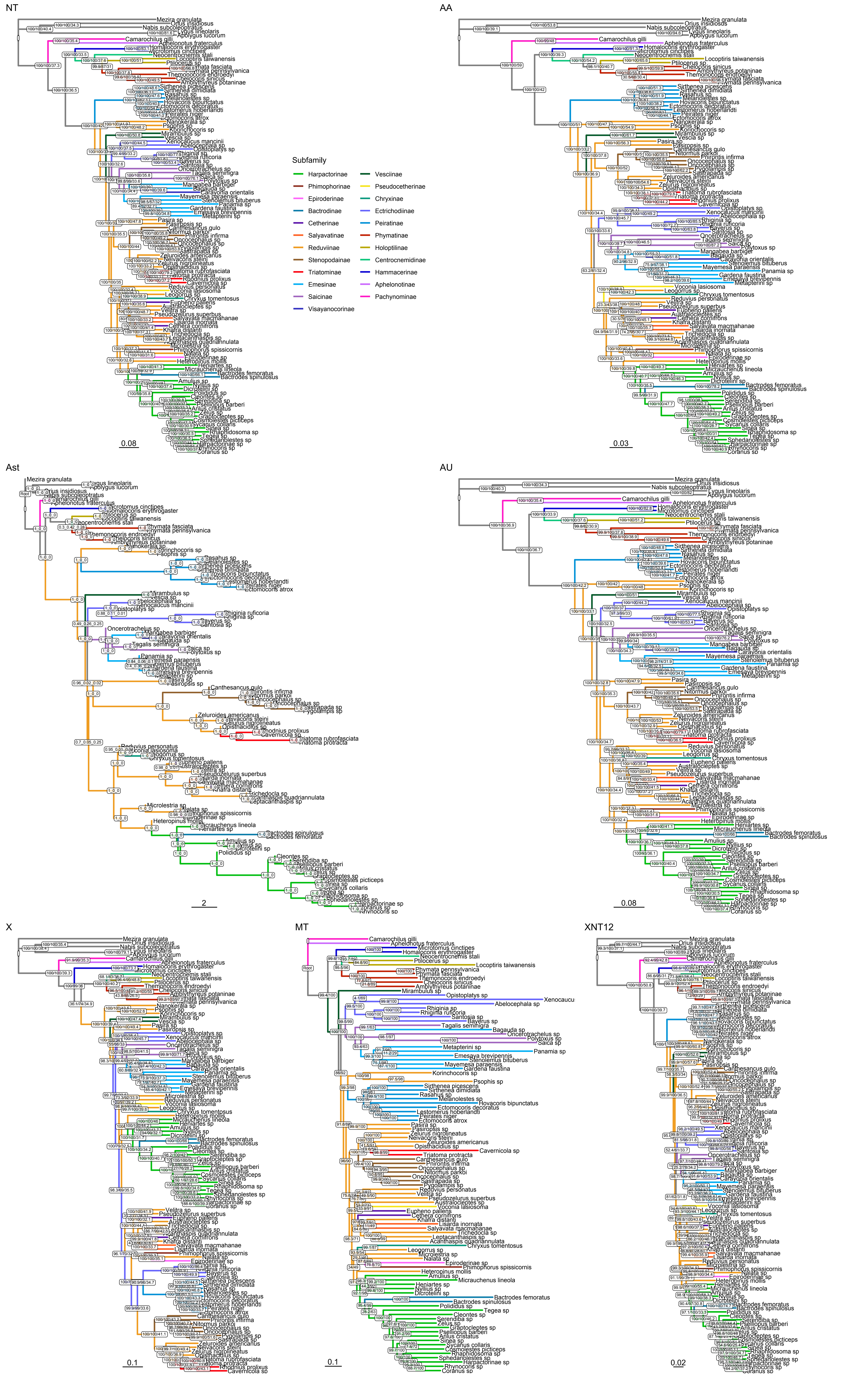

NT
AA
Ast
AU
X
MT
XNT12
Subfamily
Harpactorinae
Phimophorinae
Epiroderinae
Bactrodinae
Cetherinae
Salyavatinae
Reduviinae
Stenopodainae
Triatominae
Emesinae
Saicinae
Visayanocorinae
Vesciinae
Pseudocetherinae
Chryxinae
Ectrichodiinae
Peiratinae
Phymatinae
Holoptilinae
Centrocnemidinae
Hammacerinae
Aphelonotinae
Pachynominae
0.08
0.03
2
0.08
0.1
0.1
0.02

Supplementary Fig S2. The distribution of various locus properties in autosomal and X chromosome loci with outliers included relative to Fig. 3.

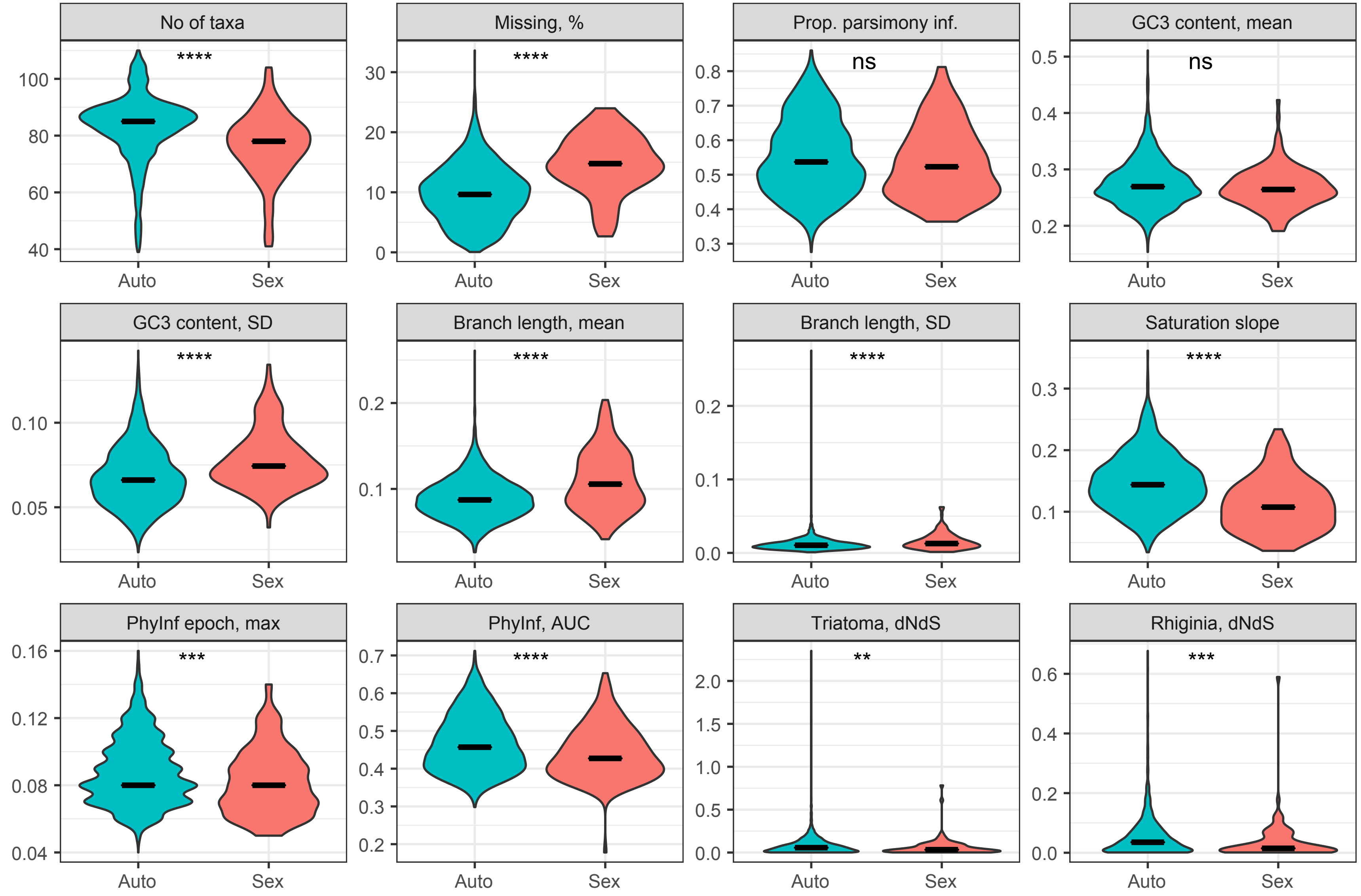

No of taxa
Missing, %
Prop. parsimony inf.
GC3 content, mean
GC3 content, SD
Branch length, mean
Branch length, SD
Saturation slope
PhyInf epoch, max
PhyInf, AUC
Triatoma, dNdS
Rhiginia, dNdS
****
ns
***
**
Auto
Sex

Supplementary Fig S3. Scatter plots showing no relationship between missing data and tree rate (a) or average branch length (b) for both autosomal and X-loci, contours show densities of each group of locus points.

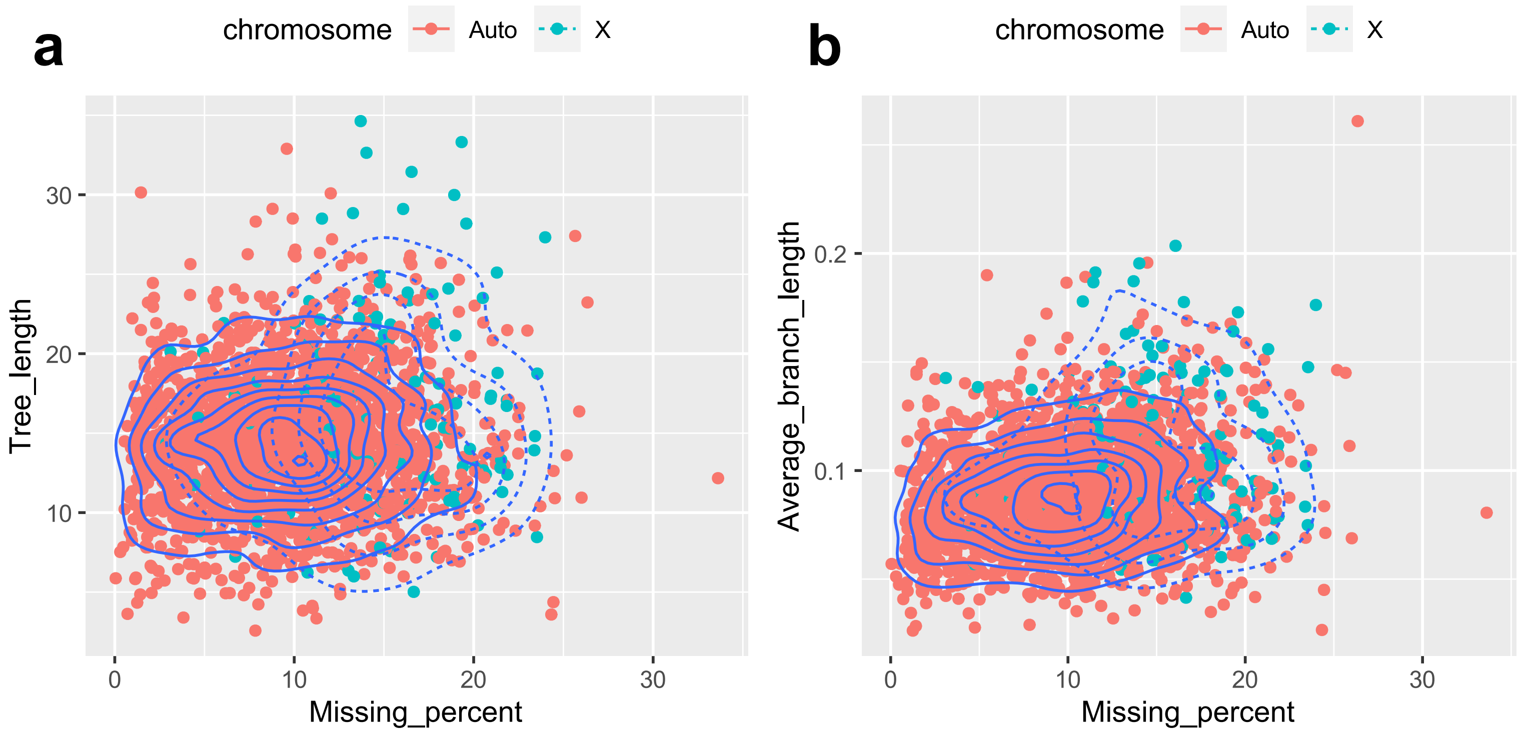
a
chromosome
Auto
X
Tree_length
Missing_percent
30
20
10
0
10
20
30
b
chromosome
Auto
X
Average_branch_length
Missing_percent
0.2
0.1
0
10
20
30

Supplementary Fig S4. IQ-TREE Binary Gene content based tree (genomes only).

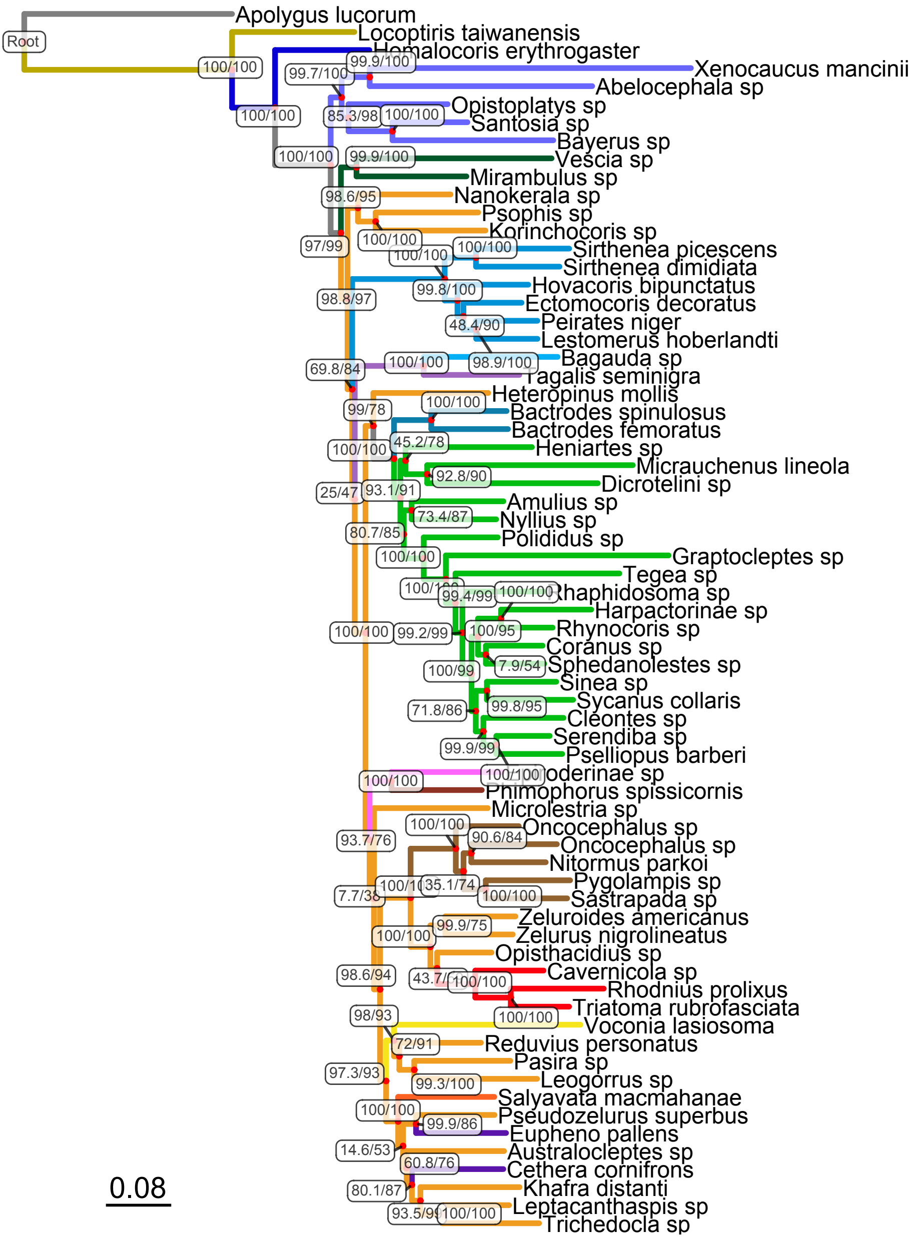

Root
Apolygus lucorum
Locoptiris taiwanensis
Homalocoris erythrogaster
Xenocaucus mancinii
Abelocephala sp
Opistoplatys sp
Santosia sp
Bayerus sp
Vescia sp
Mirambulus sp
Nanokerala sp
Psophis sp
Korinchocoris sp
Sirthenea picescens
Sirthenea dimidiata
Hovacoris bipunctatus
Ectomocoris decoratus
Peirates niger
Lestomerus hoberlandti
Bagauda sp
Tagalis seminigra
Heteropinus mollis
Bactrodes spinulosus
Bactrodes femoratus
Heniartes sp
Micrauchenus lineola
Dicrotelini sp
Amulius sp
Nyllius sp
Polididus sp
Graptocleptes sp
Tegea sp
Rhaphidosoma sp
Harpactorinae sp
Rhynocoris sp
Coranus sp
Sphedanolestes sp
Sinea sp
Sycanus collaris
Cleontes sp
Serendiba sp
Pselliopus barberi
Phimophorus spissicornis
Microlestria sp
Oncocephalus sp
Oncocephalus sp
Nitormus parkoi
Pygolampis sp
Sastrapada sp
Zeluroides americanus
Zelurus nigrolineatus
Opisthacidius sp
Cavernicola sp
Rhodnius prolixus
Triatoma rubrofasciata
Voconia lasiosoma
Reduvius personatus
Pasira sp
Leogorrus sp
Salyavata macmahanae
Pseudozelurus superbus
Eupheno pallens
Australocleptes sp
Cethera cornifrons
Khafra distanti
Leptacanthaspis sp
Trichedocla sp
100/100
99.9/100
99.7/100
100/100
85.3/98
100/100
100/100
99.9/100
98.6/95
97/99
100/100
100/100
100/100
98.8/97
99.8/100
48.4/90
69.8/84
100/100
98.9/100
99/78
100/100
100/100
45.2/78
92.8/90
25/47
93.1/91
73.4/87
80.7/85
100/100
99.4/99
100/100
100/100
99.2/99
100/95
100/99
7.9/54
71.8/86
99.8/95
99.9/99
100/100
100/100
93.7/76
100/100
90.6/84
7.7/38
35.1/74
100/100
99.9/75
100/100
98.6/94
100/100
100/100
98/93
72/91
97.3/93
99.3/100
100/100
99.9/86
14.6/53
60.8/76
80.1/87
100/100
0.08

Supplementary Fig S5. Gains and losses of 25,954 OMCL orthogroups on pruned ML tree, partitioned into AU and X subsets. Boxes at nodes show gains and losses along internal branches. Tip gains and losses annotated on the right along with specimen sex and genome completeness proxies (% of phylogenomic loci recovered and median phylogenomic locus coverage). Since the X subset includes only 809 loci and is considerably smaller than AU, color scales are independent for each subset to show relative magnitude of change per partition.

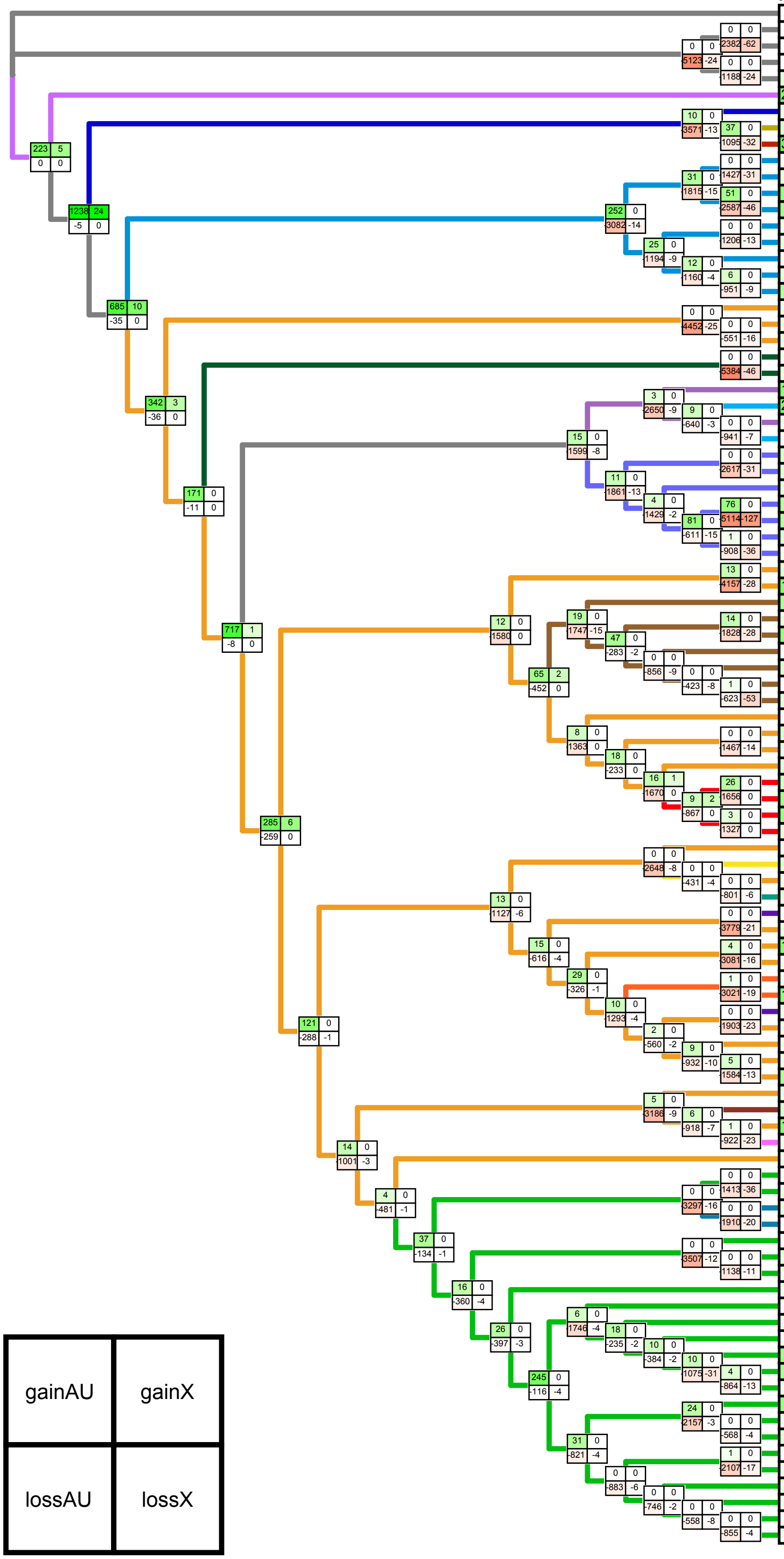

Species	gainAU	lossAU	gainX	lossX	totalAU	totalX	sex	%Nloci	MedCov
Mezira granulata	0	-8898	0	-163	8612	592			
Orius insidiosus	0	-1474	0	-32	8531	637			
Nabis subcoleoptratus	0	-1444	0	-72	8561	597			
Lygus lineolaris	0	-2909	0	-88	8290	619			
Apolygus lucorum	3	-1309	0	-55	9893	652			
Aphelonotus fraterculus	211	-8755	0	-144	9189	616			
Homalocoris erythrogaster	1	-3268	0	-29	12138	742	♀	84	-0.26
Locoptiris taiwanensis	5	-1787	0	-44	12565	695	♂	78	-0.26
Phymata pennsylvanica	396	-2808	0	-130	11935	609			
Sirthenea picescens	0	-1572	0	-65	12003	669	♂	88	-0.22
Sirthenea dimidiata	2	-1367	0	-29	12210	705	♂	89	-0.17
Rasahus sp	91	-1624	0	-66	10933	653			
Melanolestes sp	68	-1994	0	-34	10540	685			
Hovacoris bipunctatus	1	-1045	0	-27	13367	731	♂	94	-0.11
Ectomocoris decoratus	2	-1438	0	-12	12975	746	♂	94	-0.21
Lestomerus hoberlandti	0	-1602	0	-20	12867	747	♀	94	-0.18
Peirates niger	3	-777	0	-20	12750	738	♂	93	-0.07
Ectomocoris atrox	32	-3402	0	-72	10154	686			
Nanokerala sp	1	-3141	0	-16	12330	756		94	-0.14
Psophis sp	2	-950	0	-11	13971	745	♂	94	-0.29
Korinchocoris sp	2	-2338	0	-78	12583	678	♂	86	-0.23
Mirambulus sp	1	-1890	0	-70	12809	681	♂	89	-0.13
Vescia sp	2	-2064	0	-45	12636	706	♀	71	-0.18
Oncerotrachelus sp	151	-6225	0	-116	10486	665			
Metapterini sp	210	-4295	0	-127	11844	651			
Tagalis seminigra	1	-1742	0	-22	13247	749	♂	94	-0.19
Bagauda sp	0	-1900	0	-21	13088	750		93	-0.14
Xenocaucus mancinii	6	-2127	0	-49	12619	697		56	-0.24
Abelocephala sp	3	-2752	0	-61	11991	685		59	-0.1
Opistoplatys sp	2	-3605	0	-38	12329	737		91	-0.06
Rhiginia sp	29	-1729	0	-65	8664	568			
Rhiginia ruficoria	30	-1132	0	-28	9262	605			
Bayerus sp	5	-1718	0	-145	12782	579	♂	76	-0.1
Santosia sp	1	-1725	0	-18	12771	706	♂	93	-0.15
Pasira sp	4	-928	0	-25	14181	751		94	-0.06
Pasiropsis sp	156	-3554	0	-89	11707	687			
Canthesancus gulo	41	-8161	0	-251	9014	540			
Nitormus parkoi	2	-1018	0	-43	14068	718	♂	91	-0.18
Pnirontis infirma	99	-3411	0	-152	11772	609			
Oncocephalus sp	0	-2651	0	-57	13391	723	♀	85	-0.14
Oncocephalus sp	4	-2158	0	-41	13465	731	♀	92	-0.21
Pygolampis sp	0	-1416	0	-70	13581	649	♂	85	-0.26
Sastrapada sp	2	-1352	0	-114	13647	605	♂	81	-0.15
Zeluroides americanus	0	-4186	0	-22	13321	784		94	-0.18
Neivacoris steini	5	-1778	0	-148	14052	644	♀	74	-0.03
Zelurus nigrolineatus	2	-2082	0	-15	13745	777	♂	94	-0.21
Opisthacidius sp	3	-2044	0	-12	13597	795	♀	96	-0.15
Triatoma rubrofasciata	7	-1403	0	0	11754	809			
Triatoma protracta	62	-2125	0	-82	11087	727			
Rhodnius prolixus	71	-2083	0	0	11444	809			
Cavernicola sp	2	-987	0	-17	12471	792	♀	94	-0.19
Reduvius personatus	1	-3484	0	-19	13405	770	♀	95	-0.13
Voconia lasiosoma	4	-3256	0	-173	13205	612	♂	73	-0.08
Leogorrus sp	1	-1533	0	-28	14124	751	♀	88	-0.18
Chryxus tomentosus	3	-2290	0	-43	13369	736	♀	87	-0.13
Eupheno pallens	0	-2044	0	-15	13112	757	♀	94	-0.27
Australocleptes sp	2	-1172	0	-25	13986	747		94	-0.18
Velitra sp	153	-4176	0	-124	11538	652			
Pseudozelurus superbus	1	-2111	0	-28	13451	748	♂	94	-0.28
Salyavata macmahanae	1	-1446	0	-25	12890	744	♀	94	-0.07
Lisarda inornata	119	-3866	0	-128	10588	641			
Cethera cornifrons	1	-1747	0	-23	13148	740		92	-0.11
Khafra distanti	1	-1227	0	-99	13668	664	♂	88	-0.09
Trichedocla sp	1	-1380	0	-50	14495	726		93	-0.08
Leptacanthaspis sp	1	-784	0	-31	13512	732		94	-0.05
Acanthaspis quadriannulata	36	-5020	0	-154	9311	609			
Microlestria sp	2	-2646	0	-31	13838	760		95	-0.16
Phimophorus spissicornis	5	-2661	0	-22	12914	762		94	-0.12
Nalata sp	139	-3291	0	-116	11497	645			
Epiroderinae sp	1	-1390	0	-33	13260	728	♂	93	-0.22
Heteropinus mollis	1	-5740	0	-62	13447	737	♂	88	-0.12
Heniartes sp	0	-1432	0	-22	12947	724	♀	84	-0.12
Micrauchenus lineola	1	-2595	0	-174	11785	572		67	-0.23
Bactrodes femoratus	2	-1316	0	-20	12568	742		93	-0.09
Bactrodes spinulosus	0	-1400	0	-19	12482	743		92	-0.1
Amulius sp	0	-2080	0	-10	13158	772		94	-0.1
Nyllius sp	2	-1529	0	-17	12573	754		94	-0.09
Dicrotelini sp	1	-2120	0	-66	11981	705	♀	69	-0.13
Polididus sp	1	-5340	0	-48	13035	743	♀	93	-0.27
Cleontes sp	0	-3812	0	-32	12951	751		95	-0.1
Serendiba sp	1	-2873	0	-30	13674	751	♂	96	-0.21
Pselliopus barberi	0	-2087	0	-19	14085	760	♂	95	-0.2
Arilus cristatus	70	-4242	0	-147	10935	601			
Zelus sp	48	-3890	0	-60	10405	675			
Graptocleptes sp	1	-1565	0	-163	12683	572	♂	67	-0.09
Cosmolestes picticeps	75	-4375	0	-143	11280	637			
Sycanus collaris	1	-1603	0	-62	13410	714	♂	88	-0.25
Sinea sp	4	-1223	0	-13	13793	763	♀	95	-0.19
Rhaphidosoma sp	0	-1130	0	-13	13594	747	♂	93	-0.21
Tegea sp	1	-2444	0	-93	12281	667	♂	82	-0.3
Sphedanolestes sp	2	-2478	0	-24	13608	751	♀	95	-0.29
Harpactorinae sp	1	-1938	0	-36	13589	731	♂	93	-0.21
Rhynocoris sp	0	-917	0	-10	13754	753	♀	94	-0.11
Coranus sp	1	-1457	0	-57	13215	706	♂	93	-0.21

Supplementary Fig S6. Panel (a) shows a tree inferred from aligned trimmed sequences of OBP13 (orthogroup 31851), OBP14 (orthogroup 8651), and OBP23 (orthogroup 8645) gene clusters; only OBP13 was inferred to be Triatominae-specific. Panel (b) features a UPGMA tree inferred from unaligned sequences of AGO2-like gene clusters from orthogroups 26369 (green), 4180 (purple), 2404 (red), and 3589 (turquoise), showing a Harpactorine-specific expansion. Several sequences (7 on S6a and 11 on S6b) have been deemed to be misassigned and were resigned (in color) to the appropriate OG based on more accurate phylogenetic results.

a

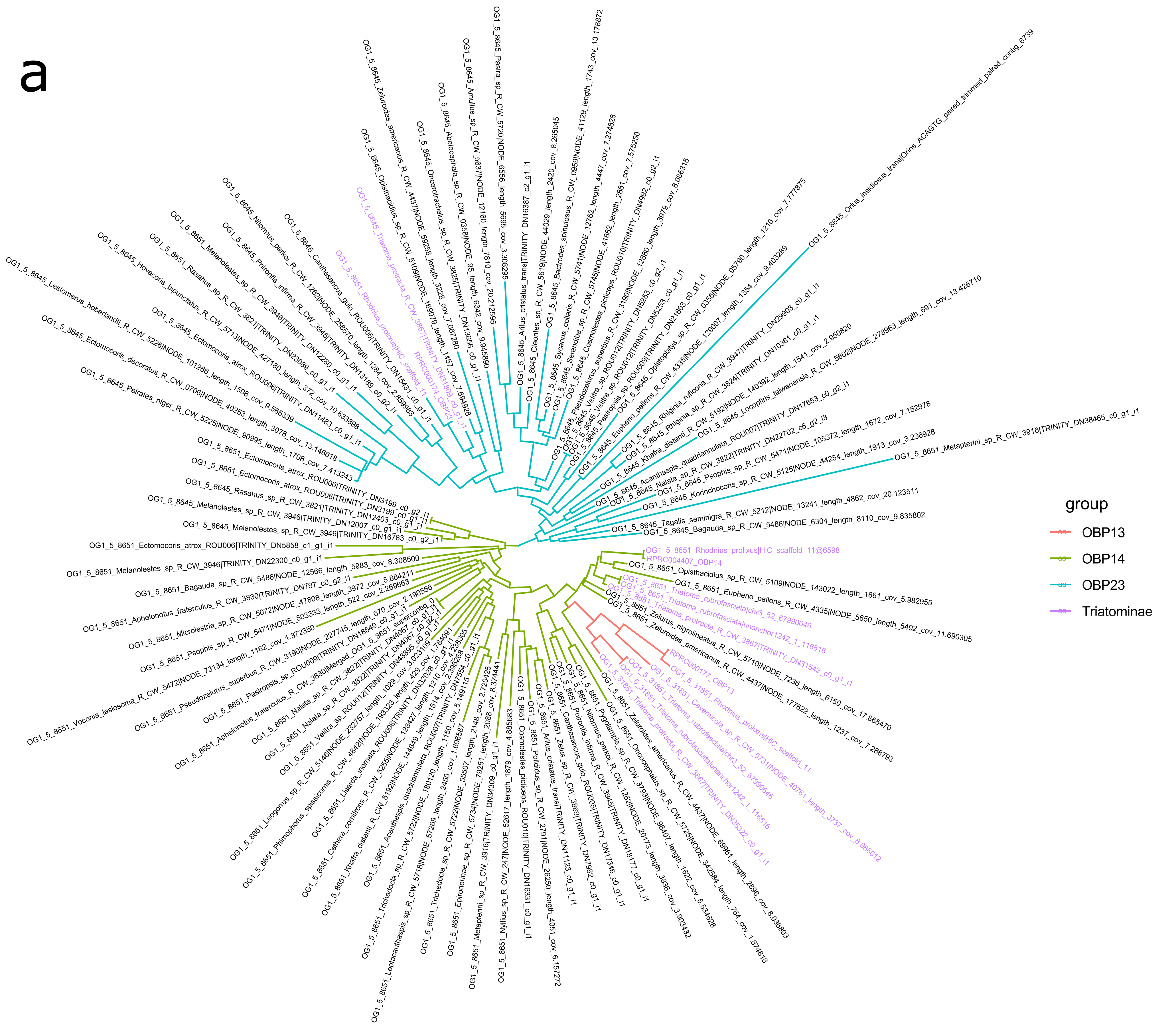

b

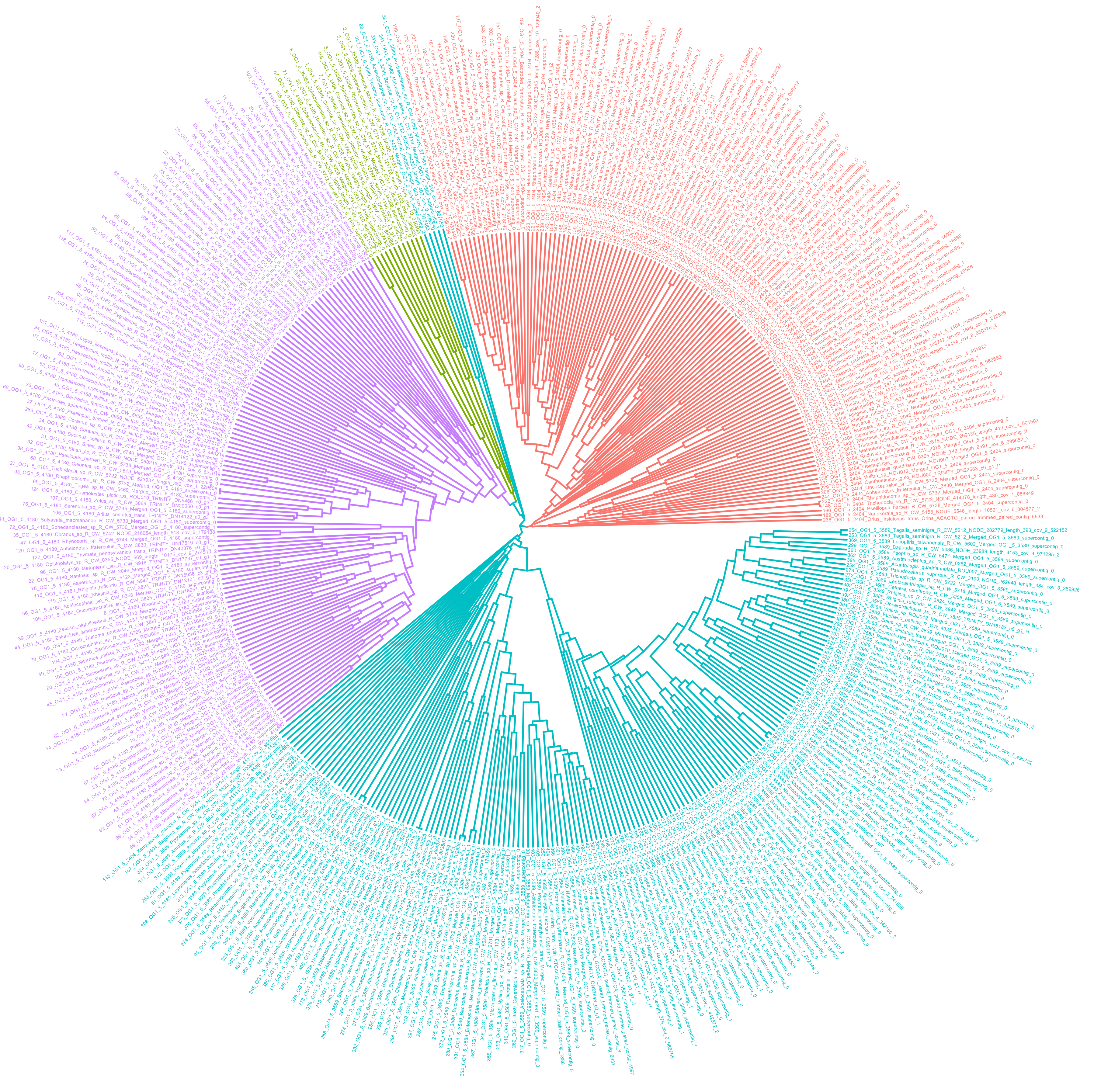

Supplement: msad168_Supplementary_Data [file msad168_supplementary_data.zip › suppl_figs.pdf]
